# Conditional Expression of Wnt9b in *Six2*-Positive Cells Disrupts Stomach and Kidney Function

**DOI:** 10.1371/journal.pone.0043098

**Published:** 2012-08-17

**Authors:** Susan M. Kiefer, Lynn Robbins, Michael Rauchman

**Affiliations:** 1 Department of Internal Medicine, Saint Louis University, St. Louis, Missouri, United States of America; 2 Department of Biochemistry and Molecular Biology, Saint Louis University, St. Louis, Missouri, United States of America; 3 Nephrology Division, John Cochran Veterans Affairs Medical Center, St. Louis, Missouri, United States of America; National Cancer Center, Japan

## Abstract

During kidney development, canonical Wnt signaling activates differentiation, while the transcription factor Six2 maintains the progenitor pool. These opposing signals help to regulate nephron formation and ensure the full complement of nephrons are formed. Since these two factors control differing fates in kidney mesenchyme, we hypothesized that overexpression of *Wnt9b* in *Six2*-expressing cells would disrupt kidney formation and may alter cell differentiation decisions in other tissues. We created a transgenic mouse that conditionally expressed the canonical Wnt ligand in the developing kidney, *Wnt9b*. The transgene is activated by cre recombinase and expresses GFP. We first tested its biological activity using *Hoxb7-cre* and found that transgenic Wnt9b was capable of inducing differentiation genes and of rescuing kidney development in *Wnt9b^−/−^* homozygous deficient mice. In contrast, expression of *Wnt9b* in cells using *Six2-cre* caused gastrointestinal distress and severe renal failure in adult mice. Transgenic kidneys had numerous cystic tubules and elevated creatinine values (0.652±0.044) compared to wild-type mice (0.119±0.002). These animals also exhibited a malformed pyloric sphincter, duodenogastric reflux, and a transformation of the distal stomach into proximal fate. The gene expression changes observed for the *Wnt9b:EGFP* transgene were compared to a stabilized β-catenin allele to determine that Wnt9b is activating the canonical Wnt pathway in the tissues analyzed. These results demonstrate that expression of *Wnt9b* in *Six2*-positive cells disrupts cell fate decisions in the kidney and the gastrointestinal tract.

## Introduction

The canonical Wnt signaling pathway is a key regulator of cell fate, cell proliferation, and cell adhesion during development and throughout adulthood. Numerous positive and negative mediators have been identified that work together to achieve tight regulation of β-catenin signaling. The central paradigm is that cytoplasmic β-catenin is sequestered in a destruction complex, phosphorylated by GSK3β, and targeted to the ubiquitin destruction pathway in the absence of a Wnt signal. Wnt binding inhibits phosphorylation and destruction of β-catenin, allowing it to translocate to the nucleus to affect target gene expression with TCF/LEF factors (reviewed by MacDonald et al. [1]). Numerous mouse models have been created to study this pathway including both gain and loss of function alleles of Wnts, Wnt receptors, β-catenin, and the TCF/LEF transcriptional regulators. These models have uncovered essential roles for canonical Wnt signaling in bone, hair, intestine, blood, and cancer [2]. An emerging theme from these studies is that many biological processes are acutely sensitive to the strength of canonical Wnt signaling.

This tight regulation of canonical Wnt signaling is essential for kidney development. During the initial stages of kidney formation, Wnt9b is secreted from the ureteric bud and signals to progenitor cells in the cap mesenchyme to induce nephron differentiation. Treatment with lithium chloride or activation of β-catenin can induce nephron differentiation in the absence of the ureteric bud and Wnt9b, suggesting that an increase in canonical Wnt signaling is sufficient for this inductive event [3], [4]. Some cap mesenchyme cells proliferate to maintain the progenitor cells that are required for subsequent iterations of this process [5]. This balance between progenitor maintenance and the induction of differentiation is of paramount importance to ensure that the kidney forms the full complement of nephrons [5], [6], [7], [8], [9].

Several factors expressed in the cap mesenchyme have been shown to antagonize Wnt-stimulated differentiation including the transcription factor *Six2*. Deletion of *Six2* results in ectopic differentiation, depletion of cap mesenchyme progenitors, and kidney agenesis [9]. Although the exact mechanism is not yet known, a hypothesis has been proposed whereby high levels of canonical Wnt signaling drive the commitment to differentiation whereas high Six2 activity in the same cells maintains the progenitor fate [7]. We have created a new mouse model to conditionally overexpress Wnt9b to activate the canonical Wnt signaling pathway. We have used this model to test whether increased Wnt9b activity is sufficient to disrupt the balance between progenitors and differentiation in the cap mesenchyme.

A second site where both Six2 activity and Wnt signaling play an important role is the pyloric sphincter. The pyloric sphincter is formed at the distal end of the stomach and functions as a valve to enable proper digestion of food prior to its entry into the duodenum. Dysfunction of the sphincter can result in reflux of duodenal contents into the stomach posing an increased risk of gastric metaplasia and cancer [10], [11]. This region is also the site of congenital anomalies including the rare disorder primary duodenogastric reflux and the more common birth defect pyloric stenosis [12]. *Six2* deficiency leads to agenesis of the pyloric sphincter and antagonizing Wnt activity with Sfrp1 and Sfrp2 is required to pattern the stomach [13], [14]. This led us to hypothesize that canonical Wnt activity must be tightly regulated in the pylorus similar to what has been proposed in the kidney. To test this we have used *Six2*-cre to activate the *Wnt9b* transgene in *Six2* expressing domains in kidney and stomach.

The conditional *Wnt9b* transgenic allele reported here expresses *Wnt9b* and GFP when activated by cre recombinase. It is biologically active and capable of rescuing kidney formation in *Wnt9b^−/−^* embryos. Overexpression of *Wnt9b* in the kidney ureteric bud is capable of inducing genes associated with differentiation of cap mesenchyme, but does not undergo mesenchymal-to-epithelial transition (MET) to produce morphologically distinct vesicle structures. *Wnt9b* transgene activation in *Six2*-positive cells causes kidney cysts and a transformation of the distal stomach regions into proximal stomach fate. Comparison of *Wnt9b* transgenics to an allele that produces stabilized β-catenin protein demonstrates dose-responsive gene changes and suggests that these strains represent an allelic series that should be valuable for modulating canonical Wnt signaling in other tissues.

## Results

### Transgenic mice express Wnt9b when activated by cre recombinase

**Figure 1:**
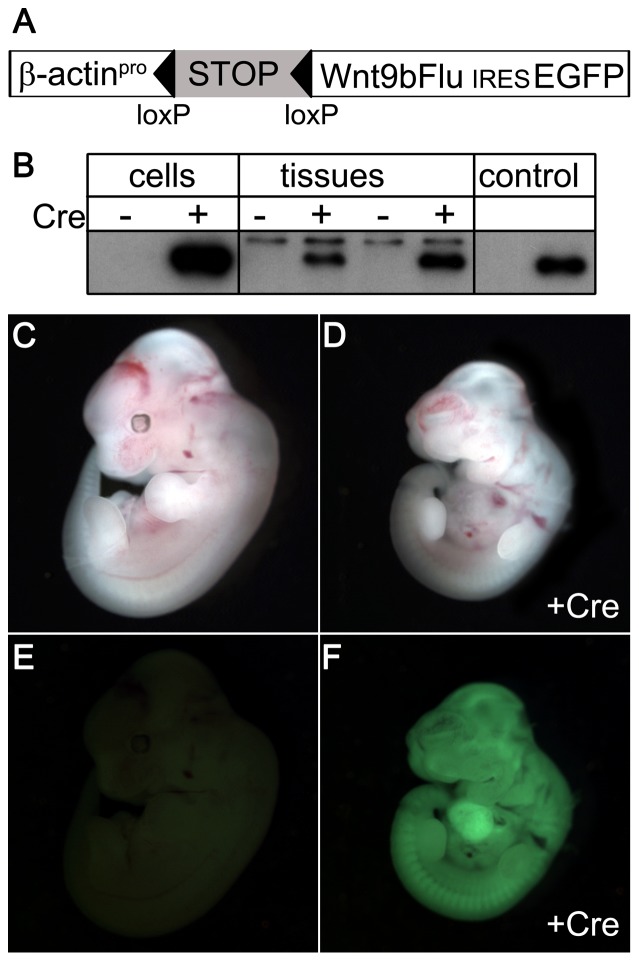
Transgene and GFP expression depends on cre in cells and tissues. (A) Schematic of the transgene construct showing the *β-actin* promoter (β-actin^pro^), the stop cassette (STOP) flanked by lox P sites (triangles), the epitope-tagged *Wnt9b* cDNA (Wnt9bFLU), an internal ribosomal entry site (IRES) and green fluorescent protein cDNA (GFP). (B) Epitope-tagged Wnt9b protein is expressed when the transgene is introduced into Cos-1 cells (lanes 1, 2) and E11.5 transgenic embryos (lanes 3–6) only when cre recombinase is present (cre +). There is a higher molecular weight background band present in the tissue lysates that is not altered by the presence of cre. Lane 7 is empty. A positive control vector that lacks the lox-STOP cassette expresses Wnt9b-FLU in the absence of cre (lane 8). Brightfield (C–D) and fluorescence (E, F) images of *Wnt9b* transgenic E11.5 embryos with (D,F) and without (C,E) *β-actin*-cre demonstrate GFP expression and delayed embryogenesis for the *Wnt9b* ^tg/+^, *β-actin*-cre^tg/+^ embryos.

The Wnt9b transgene was constructed with a lox-STOP cassette [15] to create a conditionally active Wnt9b that depends on cre recombinase for expression (Fig. 1A). Once activated, the transgene-expressed Wnt9b is distinguishable from endogenous Wnt9b because it contains a C-terminal influenza hemagglutinin epitope tag and transgenic cells will exhibit GFP fluorescence due to the IRES-EGFP cassette. To test the fidelity of the transgene, we examined cre-dependent expression in cultured cells and in vivo in embryos (Fig. 1B). Epitope-tagged Wnt9b was detectable in lysates of cells co-transfected with the transgene and a cre plasmid, and not in transfections of the transgene alone. Similarly, lysates from embryos that were positive for both Wnt9b and β-actin-cre transgenes contained Wnt9b-FLU whereas single Wnt9b transgenics did not. GFP fluorescence was observed in Wnt9b/β-actin-cre double positive embryos and not in Wnt9b littermates (Fig. 1C–F). The expression level of transgenic Wnt9b correlated with intensity of GFP fluorescence for each founder line. (Fig. 1D). Four independent founder lines were unable to produce live β-actin-cre^tg/+^, Wnt9b^tg/+^ double heterozygous pups, demonstrating that ubiquitous elevated Wnt9b expression is embryonic lethal. Analysis of embryonic stages revealed that β-actin-cre^tg/+^, Wnt9b^tg/+^ double heterozygous embryos were detected at less than expected Mendelian frequencies at E11–E12. A single double heterozygous embryo was observed from a founder with very high expression (Fig. 1A, n = 23, expected frequency 25%) and 16% were observed from a different founder (n = 25). Wnt9b/ß-actin-Cre double transgenic embryos had smaller hearts with pooling of blood, suggesting cardiac insufficiency as the cause of death.

### Transgenic Wnt9b is biologically active

**Figure 2:**
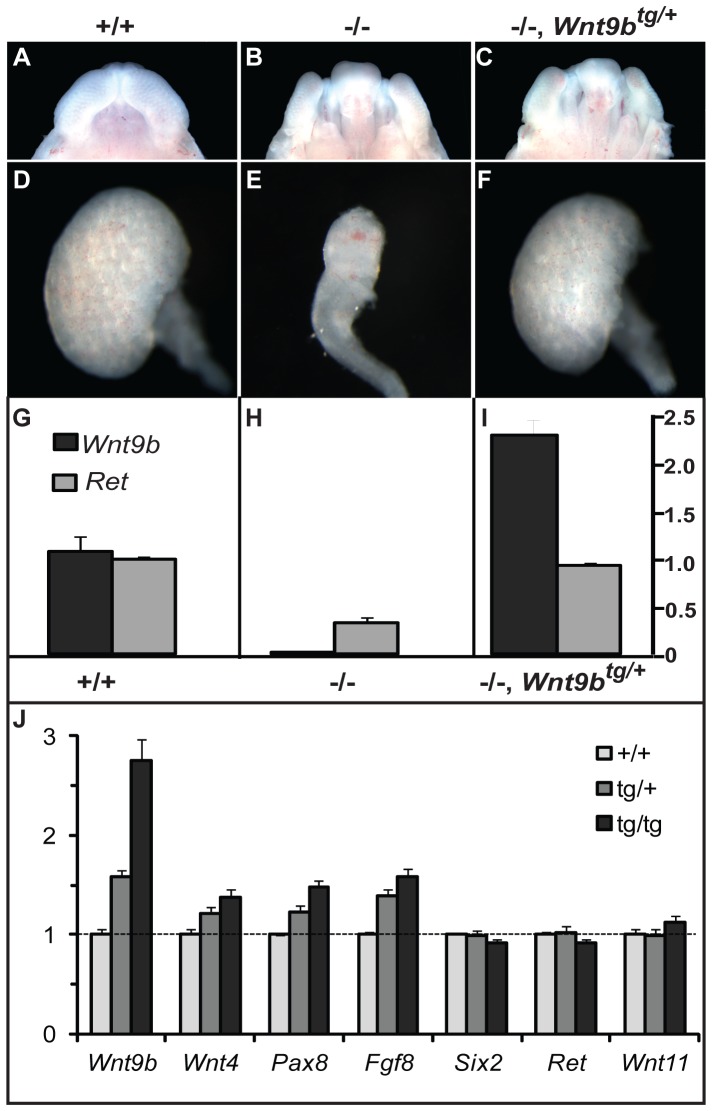
Transgenic *Wnt9b* is biologically active and can induce expression of renal vesicle genes. (A–F) Transgenic *Wnt9b* can rescue kidney formation in *Wnt9b^−/−^* E14.5 embryos. *Wnt9b^−/−^* embryos exhibit cleft palate (B) and kidney agenesis (E). Kidney-specific expression of the *Wnt9b* transgene with *Hoxb7*-cre rescues kidney formation (F), but not palate closure (C) in the *Wnt9b* ^−/−^ mutant. (G–I) Quantitative PCR analysis shows that the *Wnt9b* transgene is expressed 2.3-fold higher than wild-type *Wnt9b*. J) *Wnt9b* and the renal vesicle genes (*Wnt4*, *Pax8*, *Fgf8*) are increased in a dose-dependent fashion in wild-type (*Hoxb7-cre^tg/+^*), hemizygous (*Hoxb7-cre^tg/+^*, *Wnt9b^tg/+^*), and homozygous (*Hoxb7-cre^tg/+^*, *Wnt9b^tg/tg^*) E12.5 embryonic kidneys (p<0.05 for all pairwise comparisons except *Wnt4* hemizygous vs. homozygous (p = 0.08). This is consistent with Wnt9b's role in signaling from the ureteric bud to induce renal vesicle differentiation in the mesenchyme. A gene expressed in the metanephric mesenchyme and not in renal vesicles (*Six2*) is unaffected by the *Wnt9b* transgene. Ureteric bud markers *Ret* and *Wnt11* are also unchanged.

To determine if transgenic Wnt9b is biologically active, we tested if it was capable of replacing wild-type *Wnt9b* during kidney development. *Wnt9b^−/−^* embryos exhibit high penetrance cleft palate and renal agenesis phenotypes (Fig. 2). Activation of transgenic Wnt9b in the ureteric bud with *Hoxb7*-cre rescued kidney formation, but not palate closure at E14.5 in all *Hoxb7*-cre^tg/+^, *Wnt9b* ^−/−^, *Wnt*9b^tg/+^ embryos (n = 7). This kidney-specific rescue confirms that transgenic Wnt9b expression is dependent on cre activation, because the palate does not express *Hoxb7*-cre. Quantitative PCR of wild-type, *Wnt9b^−/−^* and *Wnt9b^−/−^*, Wnt9b^tg/+^ transgenic kidneys revealed that transgenic *Wnt9b* was expressed at 2.3- fold higher levels than the wild-type gene, while expression of *Ret* was unchanged (Fig. 2 G–I). The ability of transgenic Wnt9b to signal to the mesenchyme to replace wild-type *Wnt9b* activity confirms that it is biologically active in vivo.

A wealth of data has shown that Wnt9b and β-catenin signaling are critical inductive signals that initiate differentiation of cap mesenchyme into epithelialized renal vesicles [4], [8], [16]. Wnt9b produced in the ureteric bud activates the canonical Wnt signaling pathway in the adjacent mesenchyme and results in the expression of genes that regulate mesenchymal to epithelial transition (MET). Therefore, we tested whether overexpression of *Wnt9b* in the ureteric bud would signal to the mesenchyme to activate MET gene expression and to promote increased induction of nephrons. *Wnt9b* was overexpressed in the ureteric bud using *Hoxb7*-cre:EGFP. E12.5 embryos were genotyped by quantitative PCR analysis to determine if they were hemizygous or homozygous for the *Wnt9b* transgene and the kidneys were collected for gene expression analysis. We detected a dose-dependent increase in *Wnt9b* that was accompanied by a proportional activation of expression of the Wnt9b target genes, *Wnt4*, *Pax8*, and *Fgf8* in three independent pools of transgenic kidneys (Fig. 2J). *Six2* is expressed in undifferentiated (cap) mesenchyme and is downregulated as vesicle differentiation ensues [17]. *Six2* mRNA expression is unaffected in *Hoxb7*-cre, *Wnt9b* double transgenics. Similarly, the ureteric bud genes, *Ret* and *Wnt11*, are not significantly altered by overexpression of *Wnt9b*. The specific upregulation of genes in the mesenchyme confirms that transgenic Wnt9b is capable of signaling from the ureteric bud to induce genes important for MET, however, no histological evidence of ectopic epithelialized renal vesicles was evident (data not shown). This suggests that upregulation of *Wnt9b* in the ureteric bud is sufficient for gene activation in the mesenchyme, but not for morphological differentiation of renal vesicles.

### Misexpression of Wnt9b disrupts kidney function and pyloric sphincter formation by activating canonical β-catenin signaling

**Figure 3:**
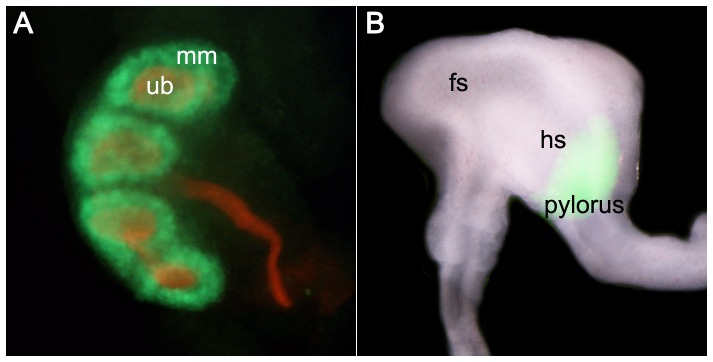
Whole mount expression pattern of *Six2*-cre in E12.5 kidney and stomach. (A) *Six2*-cre (GFP, green) is expressed in the cap metanephric mesenchyme (mm) surrounding the ureteric bud tips. Wnt9b expression and canonical Wnt signaling is highest in the ureteric bud which is labeled with cytokeratin (red). (B) In the stomach, *Six2*-cre is expressed in the hindstomach and pyloric region. Canonical Wnt signaling activity is downregulated in this region [13].

The paradigm emerging in the kidney is that there is a delicate balance between progenitor self renewal and activation of differentiation [6], [7], [9]. The Wnt9b/β-catenin pathway is required for differentiation and Six2 suppresses differentiation at the initial step of commitment. Therefore, we hypothesized that the ability of Six2 to promote fate decisions opposite from those activated by high β-catenin activity could be a common theme in development. We tested this possibility by activation of transgenic *Wnt9b* with *Six2*-cre. The double transgenics express *Wnt9b* in the same cells that express *Six2* and could disrupt processes that are coordinately regulated in the kidney and other tissues. *Six2-cre^tg/+^*, *Wnt9b^tg/+^* mice exhibited distress at weaning and body weight measurements were reduced by 39% at four weeks (14.5±3.4 grams, p<0.001) compared to cre-negative *Wnt9b^tg/+^* littermates (23.6±0.7 grams). Phenotypic analysis revealed that this reduction in body weight was due to malformations in the gastrointestinal system and the kidney, two sites of *Six2*-cre expression (Fig. 3).

**Figure 4:**
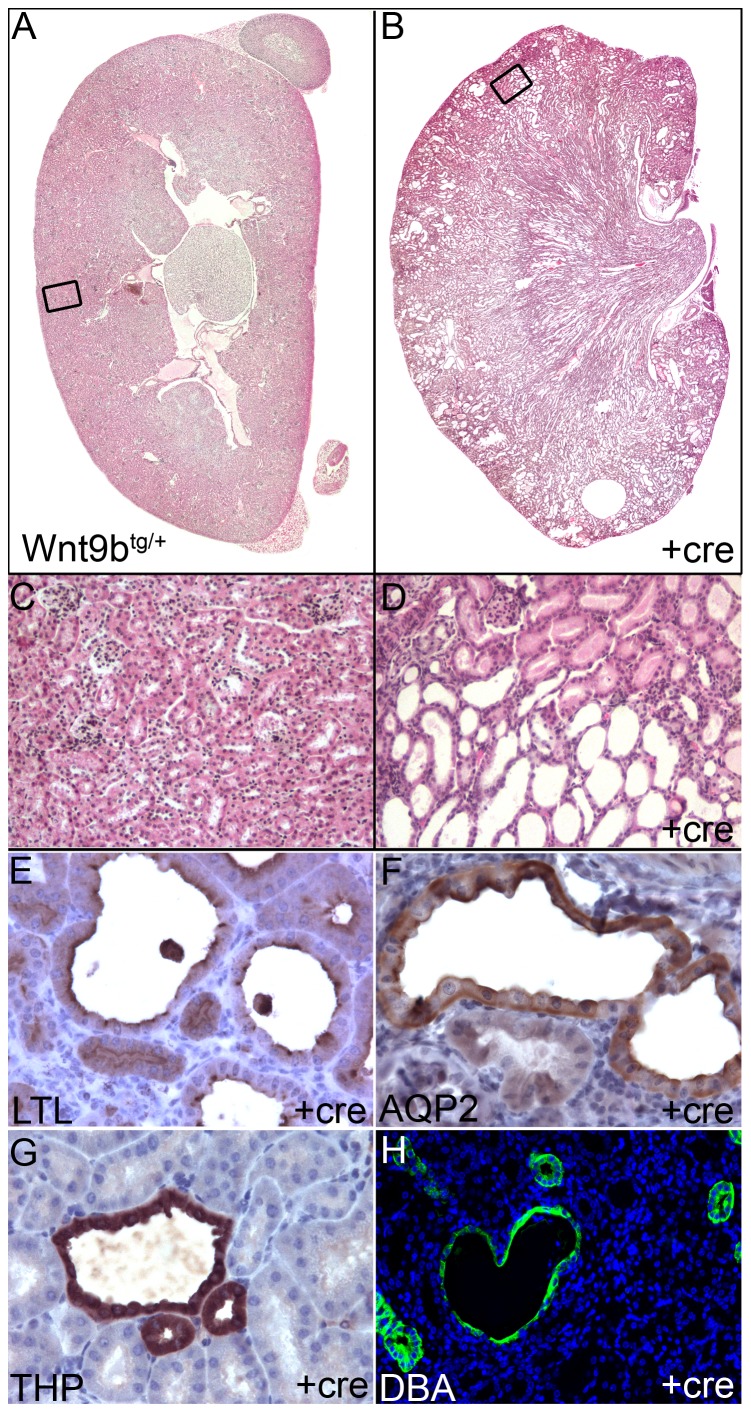
Kidney cysts in double transgenics can be found throughout all segments of the nephron. (A–D) H&E staining of adult kidneys demonstrates numerous cystic dilatations in the cortex and outer medulla in *Six2-cre^tg/+^*, *Wnt9b^tg/+^* double transgenics and a normal kidney architecture in *Wnt9b^tg/+^* controls. (E–H) Cysts were derived from proximal tubules (LTL-positive) and distal loop of Henle (THP-positive). DBA-positive and Aquaporin 2- positive collecting duct cysts were also identified in smaller number. Since *Six2*-cre is not expressed in the collecting duct, these cysts are produced through signaling from neighboring cells or secondary to dilatation of more proximal segments.

Numerous cysts of varying sizes were visible in the cortex and medulla of the double heterozygous kidneys (Fig. 4, n = 11/11). The cysts were predominantly located in the cortex and outer medulla and were of proximal tubule origin as demonstrated by LTL-staining. The cystic disease was first evident soon after birth (P7–10) in the proximal segments of the nephron (data not shown). These double heterozygous animals exhibited elevated serum creatinine levels in (0.652±0.044, p<0.005) compared to control littermates (0.119±0.002) as early as ∼3 weeks of age, indicating severe kidney failure. Older animals with advanced disease also contained cysts derived from more distal segments including the loop of Henle (Tamm-Horsfall-positive). In all animals with advanced kidney failure examined, we observed many collecting duct-derived cysts that expressed Aquaporin 2 or bound *D. Biflorus*-lectin. Since *Six2*-cre is not expressed in the collecting duct, this dilatation is likely secondary to dilatation in more proximal segments. The presence of kidney cysts in *Wnt9b* transgenic animals strengthens the conclusion that disruption of the Wnt signaling system causes cystic disease [7], [18], [19].

**Figure 5:**
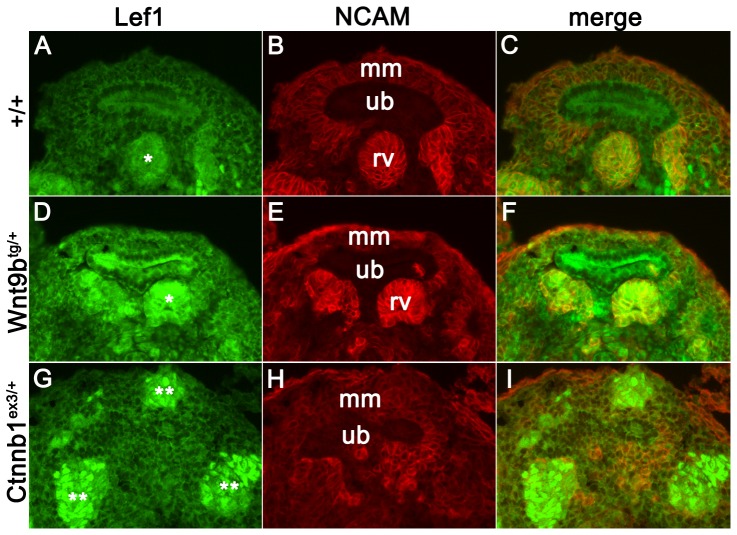
Lef1 expression exhibits dose-dependent effects in the *Wnt9b* and stabilized β-catenin alleles. (A–C) Lef1 expression (green) is low in the self-renewing cap metanephric mesenchyme cells (mm) and upregulated in the differentiating renal vesicle structures (rv). NCAM expression is also increased as the cap mm differentiates, but is not expressed in the ureteric bud (ub). (D–I) *Wnt9b* transgenic kidneys have increased Lef1 staining which is further upregulated in the ectopic vesicles seen in the stabilized β-catenin allele. Notably, not all ectopic vesicles in the stabilized β-catenin allele are NCAM positive, suggesting abnormal specification of these differentiated structures.

Although kidney cysts were not present until after birth, upregulation of canonical Wnt signaling was apparent at E12.5 when *Six2*-cre is initially expressed. Lef1 protein expression was increased in pre-tubular aggregates and renal vesicle structures in the double transgenics and was further increased in the numerous ectopic vesicles formed in stabilized β-catenin kidneys (N = 3, Fig. 5). The dose-responsive increase in Lef1 expression supports published data that Wnt9b is acting though the canonical Wnt signaling pathway in the developing kidney [7]. The lack of ectopic vesicle structures in the *Wnt9b* transgenics suggests that the *Wnt9b* allele exhibits a moderate effect on β-catenin signaling when compared to the stabilized β-catenin allele.

**Figure 6:**
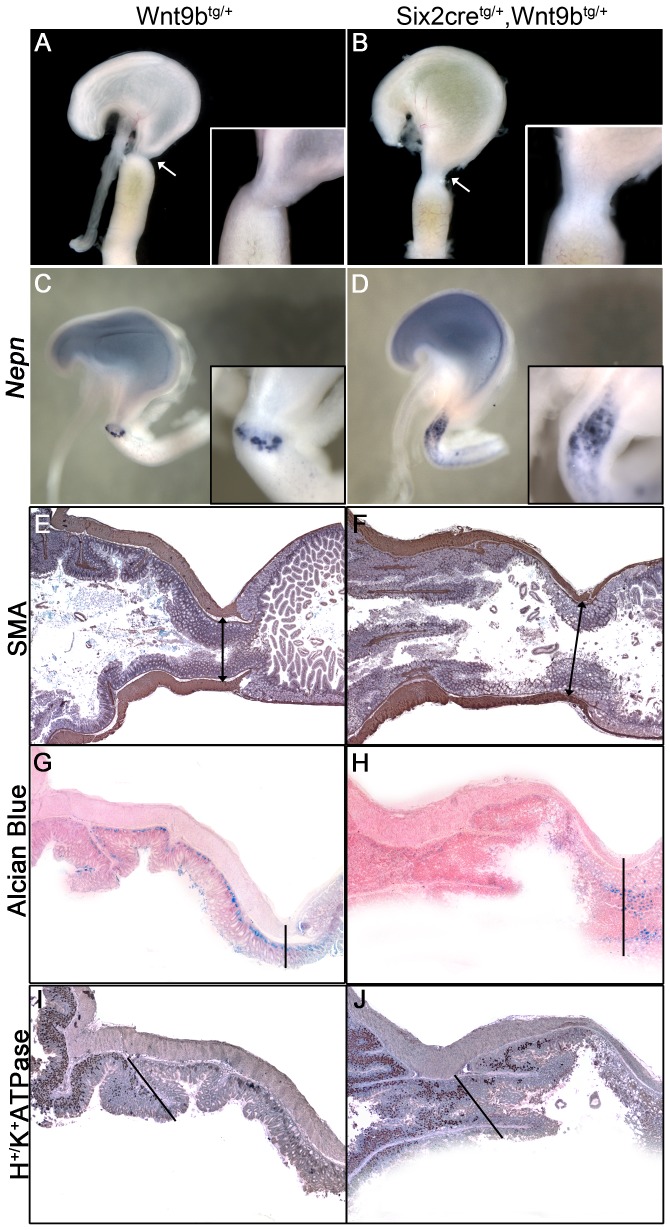
Duodenogastric reflux and pyloric sphincter abnormalities in *Six2*-cre^tg/+^, *Wnt9b* ^tg/+^ double heterozygotes. (A, B) Yellow amniotic fluid refluxed into the stomach of E18.5 double heterozygotes, but was properly retained in the intestine in control animals. A lack of constriction (arrow) and an increased diameter (inset) suggests that the reflux is due to a malformed pyloric sphincter. (C, D) Whole mount staining for *Nephrocan* mRNA delineates a thin band demarking the sphincter at E16.5 that is expanded in the double heterozygotes. (E, F) Smooth muscle actin staining in sections from control adult stomach demonstrates a thick muscular layer that leads to the sphincter. Double heterozygotes exhibit a thickened muscular layer; however it does not coalesce at the point of highest constriction. Vertical lines mark the transition from antral stomach (left side) to duodenum (right side) in E–H. (G, H) Alcian blue stains mucosal cells in the antral stomach in control sections and these cells are absent in the double heterozygotes. Mucosal cells in the intestine are stained normally. (I, J) Staining for the H^+^/K^+^ ATPase detects parietal cells in the forestomach that are excluded from the distal stomach. These cells are found throughout the distal stomach and pylorus in double heterozygotes. Sections depicted in panels I and J highlight the transition between forestomach and distal stomach (diagonal line) and do not include the duodenum.

The second phenotype contributing to reduced body weight in *Six2-cre^tg/+^*, *Wnt9b^tg/+^* double heterozygotes was gastric distress. Yellow amniotic fluid was observed in the stomach of double transgenics and not in single transgenic littermates (n = 6, Fig. 6). This indicated that duodenogastric reflux was occurring and could be due to abnormal pyloric sphincter formation. The sphincter between the stomach and the duodenum was elongated and widened in the double transgenic (Fig. 6, inset). The TGFβ antagonist, Nephrocan, which is normally expressed in a very restricted pattern at E16.5, was expanded in double transgenics, suggesting that the sphincter region was not properly constrained. This elongated morphology was supported by immunohistochemical staining of the smooth muscle layer that forms the dense muscular wall surrounding the pylorus (Fig. 6 E, F). In single transgenic controls, the thickest musculature was found where the tissue exhibits the most constriction at the sphincter (line). In the double transgenics, thick muscularity was observed, but this region was offset from the area that was the most constricted.

Mis-patterning defects were also observed by analyzing specific markers that distinguish between the proximal (H^+^/K^+^ ATPase) and the distal (Alcian blue) region of the stomach. The proximal stomach (corpus) contains H^+^/K^+^ ATPase-expressing parietal cells that are excluded from the antrum-pylorus region. Conversely, the antrum-pylorus contains mucosal cells that stain with Alcian Blue that are not found in the proximal region [20]. The double transgenics exhibited persistent H^+^/K^+^ ATPase staining and the absence of Alcian blue-positive mucosa in the antral stomach-pylorus indicating that the distal region is mis-patterned to proximal fate by *Wnt9b* expression. Alcian blue staining of goblet cells in the intestine was intact in both single and double transgenics because *Six2*-cre is not expressed in the duodenum. This mis-patterning suggests that, similar to the paradigm in the kidney, canonical β-catenin signaling specifies one fate, the forestomach region, and Six2 activity patterns the distal identity. This is supported by the expression domains of these genes since a canonical Wnt signaling reporter exhibits high activity only in the forestomach, and *Six2* is confined to the distal stomach and pylorus [13], [14].

**Figure 7:**
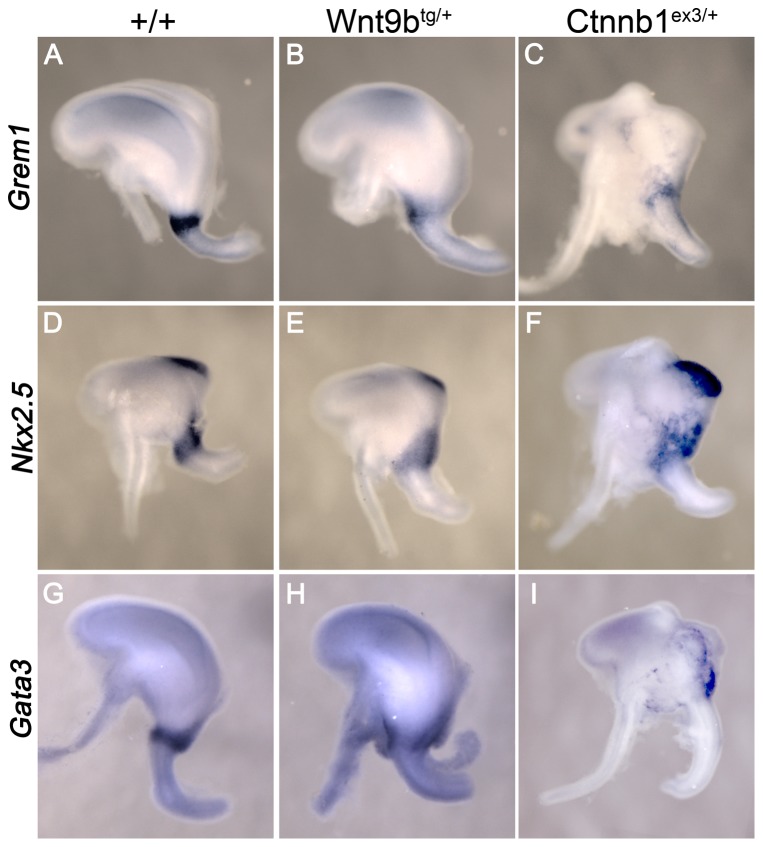
Pyloric gene expression exhibits dose-dependent effects in the *Wnt9b* and stabilized β-catenin alleles. Whole mount in situ analysis of *Grem1*, *Nkx2.5*, and *Gata3* expression in E12.5 stomachs. All three genes are restricted to the pyloric sphincter in wild-type stomachs (*Six2*-cre^tg/+^). *Grem1* and *Gata3* show decreased expression in *Wnt9b* transgenics (*Six2-cre^tg/+^*, *Wnt9b^tg/+^*) that is further downregulated in the stabilized β-catenin allele (*Six2-cre^tg/+^*, *Ctnnb1^ex3/+^*). *Nkx2.5* expression is expanded into the stomach in *Wnt9b* transgenics and further upregulated in the stabilized β-catenin allele. *Nkx2.5* expression that can be seen at the upper right of the stomach is found in the spleen on the opposite side.

If canonical Wnt signaling is responsible for the gastroduodenal reflux in *Six2-cre^tg/+^*, *Wnt9b^tg/+^* double transgenics, then the malformations observed should be recapitulated in a mouse model that expresses a stabilized β-catenin in the same domain (*Ctnnb1^ex3/+^*, [21]). After cre activation, this allele prevents the proteolytic degradation of β-catenin and would be expected to elevate canonical Wnt signaling to a higher degree than Wnt9b ligand alone. Whole mount in situ analysis of E12.5 stomachs revealed three genes, *Grem1*, *Nkx 2.5*, and *Gata3*, that are normally expressed in a thin band demarking the region that will become the sphincter (n = 8, Fig. 7). Ectopic expression of *Wnt9b* suppressed the BMP antagonist *Grem1* and the transcription factor *Gata3* in this region, and these genes were further downregulated when β-catenin signaling was increased. A dose-dependent expansion was observed for the transcription factor *Nkx2.5* in both mutants. Genes that were localized exclusively to the intestine (*villin*) or to the forestomach (*Bmp4*) were not altered in either mutant (data not shown). Increased canonical Wnt signaling in the antral stomach and pylorus was confirmed by isolating this region and performing quantitative PCR. The canonical Wnt target genes that are upregulated in the stabilized β-catenin allele, *Lef1* and *Axin2*, were also increased in the pyloric region of *Six2*-cre^tg/+^, Wnt9b^tg/+^ double transgenics [*Lef1*, 2.0±0.25 and *Axin2*, 1.4±0.06 (n = 6)]. Together, the dose-responsive gene expression changes observed in the pyloric region in the *Wnt9b* transgenics and the stabilized β-catenin embryos demonstrate that increased canonical Wnt signaling disrupts distal stomach and pyloric sphincter development.

## Discussion

These results describe the creation of a new mouse model that conditionally activates canonical Wnt signaling. The *Wnt9b* transgene is inducible upon exposure to cre recombinase and expresses GFP as a marker of induction. It also retains full biological activity since it can rescue *Wnt9b^−/−^* phenotypes in the kidney. These mice have been maintained in our colony for over a year and induce *Wnt9b* expression upon breeding to multiple different cre strains. We have utilized this novel strain to activate Wnt signaling in *Six2*-positive cells and found deleterious effects in the kidney and the stomach.

In the kidney, Wnt9b is secreted from the ureteric bud epithelium to induce the adjacent cap mesenchyme progenitor cells to initiate differentiation. In support of this model, our in vivo gain-of-function studies show that *Wnt9b* upregulates early markers of renal vesicle differentiation such as *Wnt4*, *Fgf8* and *Pax8*. However, even though renal vesicle genes are induced by the *Wnt9b* transgene, we did not observe morphological evidence of increased or ectopic renal vesicle formation, as reported for *Six2* mutants [9]. In addition to a Wnt inductive signal, factors that maintain the renal progenitor pool (e.g. *Six2*) must also be downregulated to fully elicit renal vesicle formation. *Six2* expression was not altered in our *Wnt9b* transgenic and may thus explain why the increase in Wnt9b did not result in ectopic renal vesicles. Ectopic induction of renal vesicle markers is induced by activation of β-catenin in *Six2*-positive metanephric mesenchyme using the stabilized β-catenin allele [4], therefore, the difference between these two gain-of-function experiments may relate to the degree to which they activate canonical Wnt activity. In addition, it is likely that activation of canonical signaling by Wnt9b in metanephric mesenchyme induces feedback inhibition, as noted in other Wnt responsive tissues, thereby preventing induction of the full differentiation program [22]. Recent data support this idea of feedback inhibition since Wnt9b/β-catenin signaling is required not only for the differentiation of metanephric mesenchyme, but paradoxically the same signal is also required for maintenance or expansion of the renal progenitor pool [7]. We speculate that the stabilized β-catenin, acting downstream in the pathway, may bypass these regulatory mechanisms. These possibilities could be tested in future studies in that compare downstream gene activation in renal progenitor cells using the *Wnt9b* transgene and stabilized β-catenin gain-of-function mice.

Perturbations in the canonical Wnt signaling pathway have been shown to be important in the pathogenesis of renal cystic disease (reviewed by Patel et al. [23]). Transgenic expression of a stabilized β-catenin or deficiency of APC in renal tubular epithelia leads to renal cystic disease, supporting the conclusion that ectopic canonical Wnt signaling is sufficient for cystogenesis [18], [19]. Renal failure and cystic dilatation in *Six2*-cre^tg/+^, Wnt9b^tg/+^ double heterozygotes further supports the hypothesis that upregulated canonical Wnt signaling causes cysts. Disruption of non-canonical Wnt signaling (PCP) pathway is also important in renal cyst formation [24]. Thus, it is possible that cyst formation in our model is not simply due to activation of canonical signaling, but is the result of disruption of the relative balance between canonical and non-canonical signaling. In support of this idea, the genes that are mutated in the inherited forms of cystic disease (*PKD1*, *PKD2*, *PKHD1*, *Inversin*) localize to cilia and these organelles have been recently shown to be able to regulate the balance between canonical and non-canonical Wnt signaling [25], [26].

There was a severe kidney phenotype even though *Wnt9b* mRNA expression is increased ∼2-fold in our transgene, indicating that relatively low level, persistent activation of this pathway leads to renal dysfunction. This level of upregulation of Wnt9b in the ureteric bud using *Hoxb7*-cre did not produce cysts, suggesting that cystogenesis in the collecting ducts in *Six2*-cre^tg/+^, Wnt9b^tg/+^ double transgenics is likely a secondary effect of dilated tubular segments (data not shown). In support of this, in post-natal day 7–10 animals, cystic disease is evident in only tubular segments proximal to the collecting duct. Similar to polycystic kidney disease in patients, the transgenic mice developed severe renal failure, even though most nephrons do not develop cysts. This raises the question of whether renal tubules without overt cysts are abnormal and contribute significantly to progressive functional impairment in these disorders. Recent studies demonstrate that adult renal tubule epithelia express Wnt9b, Frizzled receptors and LRP5/6 co-receptors that are capable of mediating canonical Wnt signaling in response to acute ischemic injury [27]. In this setting, Wnt signaling promotes tubule repair and regeneration. However, Wnt signaling activity has also been implicated in promoting renal fibrosis after injury, such as in the UUO model [28]. Overall, these studies highlight the importance of regulating Wnt signaling in the adult kidney to balance beneficial responses, such as repair/regeneration, and maladaptive responses leading to fibrosis and chronic organ dysfunction.

The pyloric region of the stomach represents a critical boundary that separates stomach from intestine. Sphincter closure when a food bolus enters the stomach enables proper digestion before transit to the small intestine. The molecular basis for formation of this discrete region is not well understood, though some details are emerging. These results suggest that tight control of Wnt signaling is vital for development of the pylorus. Canonical Wnt activity is detected in the fundus and body (corpus) but excluded from the distal stomach and pylorus [13]. In *Barx1*−/− mice, the stomach is small, the antral-corpus boundary is blurred and there is agenesis of the pyloric sphincter. Elegant tissue recombination studies suggest that these phenotypes result from loss of expression of the Wnt antagonists Sfrp1 and Sfrp2. However, since *Barx1* deficiency has global effects on foregut development, it was not clear if down-regulation of canonical Wnt activity is specifically required for formation of the pylorus. Our studies more directly tested this possibility by targeting ectopic Wnt activity to a restricted region just anterior to the pylorus with *Six2*-cre.

We found that ectopic Wnt activity specifically disrupts formation of the pyloric sphincter in the absence of global defects in stomach development. Thus, down-regulation of canonical Wnt activity is required to pattern the pyloric region that separates stomach from intestine. Ectopic expression of the Wnt9b ligand and stabilized β-catenin in transgenic mice could overcome the effects of secreted endogenous Wnt antagonists Sfrp1/2, consistent with the model proposed by Kim et al [13]. Similar to our results, in *Six2* mutants there is reduced expression of *Nkx2.5* and *Gremlin*, genes that are important for pyloric sphincter development in chick [14], [29]. This suggests that *Six2* and *Wnt* regulate a common genetic cascade in the presumptive pyloric sphincter. We postulate that an important function of *Six2* may be to interpret canonical Wnt signals in different developmental contexts.

Upregulated canonical Wnt signaling has also been shown to affect spleen and pancreas development [30]. In support of this, spleen and pancreas hypoplasia was also observed in *Six2*-cre, *Wnt9b* bi-transgenic mice (data not shown). Since *Six2*-cre is not expressed in these tissues, this suggests that signals from the posterior stomach mesenchyme can affect the spleen and pancreas during development. This would support the hypothesis that the pyloric region may act as an organizer for formation of these accessory organs. During development, spleen precursor cells transiently associate with the pyloric region, prior to their migration to the left of the stomach where they condense [31]. Although we did not detect *Six2*-cre expression in the spleen primordium or mesothelium, we cannot exclude that transient expression of *Six2*-cre in these regions is responsible for defects in the spleen and pancreas. Future studies are needed to directly test these possibilities.

In summary, we have developed a new mouse model that allows for temporal and spatial expression of Wnt9b. In the developing kidney, transgenic *Wnt9b* can induce early MET gene expression in *Six2*-positive renal progenitors, but it is not sufficient to activate the full differentiation program. Persistent expression of Wnt9b in *Six2*-positive cells leads to kidney cysts and severe organ failure. In the stomach, ectopic Wnt β-catenin activity in the pyloric region alters sphincter formation and distal stomach patterning. Together, these findings highlight the importance of tight control of Wnt activity and suggest that Six2 and β-catenin are involved in organ patterning in different contexts.

## Materials and Methods

### Ethics statement

The animal studies were approved by the animal care and use committee of Saint Louis University (protocol 1460) and the St. Louis VA (protocol 0305). All procedures involving animals were in compliance with institutional animal welfare regulations, standards and policies.

### Mice

*Wnt9b* transgenic mice were crossed to each of three different cre lines [32]. 1) β-actin- cre was used to express Wnt9b ubiquitously (Fig. 1, [33]) 2) *Hoxb7*-cre:EGFP was used to overexpress Wnt9b in its endogenous location in the kidney, the ureteric bud (Fig. 2, [34]), and 3) the BAC *Six2*-cre:EGFP transgene was used to express Wnt9b exogenously in the stomach and the kidney (Figs. 3, 4, 5, [35]). *Ctnnb1^ex3/+^* mice have been described previously [21] and were mated to the *Six2*-cre transgene to activate canonical Wnt signaling in the distal stomach. At least two independent founder lines were analyzed for all *β-actin*-cre and *Six2*-cre matings presented in this paper. The *Hoxb7*-cre phenotypes were verified for multiple founders in a previous publication [32]. Noon on the day of vaginal plug detection was considered E0.5.

### Activation of Wnt9b-FLU expression in cells and tissues

Cos-1 cells (ATCC CRL-1650) were plated at a density of 2×10^5^ cells per well in a six well plate and transfected using Fugene HD according to the manufacturer's instructions (Roche). 150 ng cre plasmid or control DNA was co-transfected with the *Wnt9b* transgene plasmid (2 µg/well). Cells were lysed at 48 hours in lysis buffer containing inhibitors (1% Triton X-100, 200 mm sucrose, 50 mmTris [pH 7.4], 150 mm NaCl, 1 mg/ml leupeptin, 2 mg/ml antipain, 10 mg/ml benzamidine, 1 mg/ml chymostatin, 1 mg/ml pepstatin, 24 mg/ml Pefabloc, 20 mM NaF and 2 mM sodium molybdate). A plasmid that contained a constitutively-expressed Wnt9b-FLU was used as a positive control. For tissue analysis, *Wnt9b^tg/+^* or *Wnt9b^tg/+^*, *β-actin*-cre*^tg/+^* E11.5 embryos were homogenized in the same buffer. Protein lysates were quantitated and a 7.5% SDS-PAGE gel was loaded with 3 µg of cell lysates, 50 µg of tissue lysates and 10 µg positive control sample. Western Blotting using anti-FLU antibody (12CA5 1∶2000, Roche) was performed as described [36].

### Quantitative PCR

E12.5 kidneys were collected from wild-type *Hoxb7-cre* ^tg/+^, *Wnt9b^tg/+^*, *Hoxb7-cre* ^tg/+^ or *Wnt9b^tg/tg^*, *Hoxb7-cre^tg/+^*. Twelve kidneys per group were pooled and subjected to quantitative RT-PCR as described previously [36]. Relative expression of *Wnt4*, *Pax8*, *Fgf8*, *Six2*, *Ret*, *Wnt9b* and *Wnt11* was calculated using the ΔΔCT method and normalized to the gene *Rpl19* for three independent pools of each genotype. Sample sizes were smaller for the rescue experiment where two independent pools of two wild-type kidneys, two rescued kidneys, or four mutant rudiments were assayed for *Wnt9b* and *Ret* expression levels and normalized to *Hprt* expression. Statistical significance was determined by the students t-test.

### Whole mount in situ hybridization

E12.5 stomachs were fixed in 4% PFA overnight at 4°C and stored in 100% methanol. In situ was performed as described using digoxigenin-labeled antisense probes for *Grem1* (nucleotides 1 - 551), *Nkx 2.5* (26 - 896), *Gata3* (169 - 1000), *Bmp4*(10 - 888), and *villin 1* (105 - 976) [32]. Tissues were photographed using a Leica DVC300 FX camera and M165 FC microscope.

### Analysis of stomach phenotype

E17.5 and adult stomach tissues were fixed in 10% Formalin prior to dehydration and embedding in paraffin. Five micron sections were analyzed by immunohistochemistry using antigen retrieval as described previously [32]. Anti-smooth muscle actin (Millipore ASM-1, 1∶100) and anti-H+/K+ ATPase (Alexis Biochemical 2G11, 1∶500) were used to characterize the antral stomach and pyloris. Slides were counterstained with hematoxylin and mounted in Mowiol 4–88 (Polysciences). Sections were analyzed for the presence of mucosal cells by Alcian blue staining (1% Alcian blue in 3% Acetic Acid PH 2.5) for 30 minutes at room temperature. After washing in tap water, slides were counterstained in 0.1% Nuclear Fast Red Solution and mounted in Mowiol 4–88.

### Kidney staining

Anti-THP (Santa Cruz H-135, 1∶250) and anti-Aquaporin 2 (Santa Cruz H-40, 1∶20) were used to localize Loop of Henle and collecting duct cysts on paraffin sections of bisected kidneys following antigen retrieval. LTL-biotin (Vector 1∶100) binding to proximal tubule cysts was detected by Vectastain ABC streptavidin-HRP (Vector Labs) and DAB (3,3-diaminobenzidine, Pierce) according to the manufacturer's recommended protocol. DBA-FITC (Vector, 1∶100) was used to directly label kidney collecting ducts and counterstained with Dapi. Photography of all tissue sections was performed on a Nikon 80I epifluorescence microscope using MetaMorph software.
